# Milk Macrophage Function in Bovine Leukemia Virus-Infected Dairy Cows

**DOI:** 10.3389/fvets.2021.650021

**Published:** 2021-06-17

**Authors:** Ewerton de Souza Lima, Maiara Garcia Blagitz, Camila Freitas Batista, Alexandre José Alves, Artur Cezar de Carvalho Fernandes, Eduardo Milton Ramos Sanchez, Hugo Frias Torres, Soraia Araújo Diniz, Marcos Xavier Silva, Alice Maria Melville Paiva Della Libera, Fernando Nogueira de Souza

**Affiliations:** ^1^Núcleo Aplicado à Produção e Sanidade da Glândula Mamária, Departamento de Medicina Veterinária, Centro de Ciências Agrárias, Universidade Federal da Paraíba, Areia, Brazil; ^2^Programa de Pós-Graduação em Ciência Animal, Centro de Ciências Agrárias, Universidade Federal da Paraíba, Areia, Brazil; ^3^Veterinary Clinical Immunology Research Group, Departamento de Clínica Médica, Faculdade de Medicina Veterinária e Zootecnia, Universidade de São Paulo, São Paulo, Brazil; ^4^Programa de Pós-Graduação em Saúde, Bem-estar e Produção Animal Sustentável na Fronteira Sul, Universidade Federal da Fronteira Sul, Avenida Edmundo Gaievski 1000, Realeza, Brazil; ^5^Department of Public Health, School of Health Sciences, National University Toribio Rodriguez de Mendonza of Amazonas, Chachapoyas, Peru; ^6^Laboratório de Sorologia e Imunobiologia, Instituto de Medicina Tropical, Universidade de São Paulo, São Paulo, Brazil; ^7^Parasitic and Infectious Diseases Laboratory, Animal Husbandry and Biotechnology Research Institute, Universidad Nacional Toribio Rodríguez de Mendoza, Chachapoyas, Peru; ^8^Departamento de Medicina Veterinária Preventiva, Escola de Veterinária, Universidade Federal de Minas Gerais, Belo Horizonte, Brazil

**Keywords:** enzootic bovine leukosis, deltaretrovirus, immune response, mastitis, dairy cattle

## Abstract

The implications of bovine leukemia virus (BLV) on innate and adaptive immune responses have been widely investigated; however, the effects of BLV on mammary gland immunity require further investigation. The present study investigated the viability, phagocytic capacity, and intracellular production of reactive oxygen and nitrogen species (RONS) by macrophages in milk samples from dairy cows naturally infected with BLV with or without persistent lymphocytosis (PL). No effect of BLV infection in the overall number of macrophages per milliliter and in the percentage of viable macrophages among overall milk viable cells was found. Furthermore, BLV-infected dairy cows had a higher frequency of viable milk macrophages, while healthy animals had a tendency toward a higher percentage of apoptotic milk macrophages. The percentage of milk macrophages that phagocytosed *Staphylococcus aureus* in seronegative animals was higher than that in BLV-infected dairy cows. No effect of BLV infection on the intracellular RONS production and the intensity of phagocytosis by milk macrophages was observed. Thus, this study provides new insights into the implications of BLV infections in the bovine mammary gland.

## Introduction

Bovine leukemia virus (BLV) belongs to the family *Retroviridae* of the oncogenic genus *Deltaretrovirus*, which has tropism for B cells (1–4), although, it could infect and persist in other cell types, such as macrophages (1, 2). It is the etiological agent of enzootic bovine leukosis (EBL), which is widely disseminated in dairy herds in several countries (5). For the most part, the infectious implications of EBL remain asymptomatic, and these infections are termed aleukemic (AL). However, ~20–30% of BLV-infected dairy cows have dramatic hematological changes known as persistent lymphocytosis (PL), which is characterized by a non-malignant polyclonal expansion of B lymphocytes. However, its malignant presentation is rare and results in lymphoma of B cells (2).

It is understandable that different viruses have direct implications on the function of the innate and adaptive immune systems (4–6). Hereupon, several studies have investigated the effects of BLV infection on lymphocyte subpopulations (2, 4, 5, 7, 8), functions of neutrophils (4, 6, 9), mammary epithelial cells (10–12), and monocytes (13). However, to the best of our knowledge, the effects of BLV infection on the function of milk macrophages and their viability are only speculative.

Eventually, the impact of chronic viral diseases with low lethality, especially BLV, is important when correlated with comorbidities, such as mastitis. This disease remains one of the main health problems in dairy herds, and it has a direct impact on milk quality and public health due to the transmission of pathogens and antimicrobial residues in milk (4). In this sense, some studies pointed to an association between BLV in herds with a high prevalence of mastitis (14, 15), the severity of clinical mastitis (16), and reduced milk production due to immunosuppression caused by BLV leading to opportunistic infections, which are also seen in other viral infections (17). Infection by BLV also negatively impacts the culling rate of dairy cattle, and AL animals may show decreased productivity and profitability (18). Thus, these findings raised questions about the effects of BLV on mammary gland immunity, in which macrophages are the main population present in milk from health mammary glands (19–24), thus being the first defense cells to come into contact with the pathogen that causes mastitis.

Thus, considering the importance of macrophages to the mammary gland during an infectious process, the present study investigated the phagocytic capacity and intracellular production of reactive oxygen and nitrogen species (RONS) by milk macrophages and their viability in BLV naturally infected dairy cows with or without PL.

## Materials and Methods

### Animals, Experimental Design, and Sample Collection

The present study used 57 mammary quarters of 19 Holstein dairy cows from a commercial herd at different stages of lactation. Immediately postpartum animals were not used. The following exclusion criteria were applied because of the effects of bacterial mastitis pathogens on mammary gland immunity (4): (1) udder quarters with clinical mastitis detected by the strip cup test and clinical examination; (2) bacteriologically positive quarters; and (3) udder quarters with high somatic cell count (SCC) (>200,000 cells mL^−1^). All dairy cows were in their second or third lactation.

Blood sera from all animals were tested for BLV using an agar gel immunodiffusion assay (AGID) (Tecpar®, Curitiba, Brazil) and ELISA (cat. n. 284-5, VMRD Pullman Inc., Pullman, WA, USA) using the gp51 antigen. The animals were divided into three groups according to the results of the serological tests and the leukogram: (1) eight healthy dairy cows (24 mammary quarters) seronegative for BLV and without hematological alterations (25); (2) six BLV-infected dairy cows (16 mammary quarters) without hematological alterations (25), so-called AL; and (3) five BLV-infected dairy cows (17 mammary quarters) with PL. The BLV-infected cows were classified as having PL when their lymphocyte counts exceeded 1 × 10^4^ μL^−1^ and their leukocyte counts exceeded 1.5 × 10^4^ μL^−1^, as established by Thurmond et al. (26).

One hundred ten days after the first blood collection for the serodiagnosis of EBL, additional blood samples were collected for hematological examinations and serodiagnosis for the BLV to confirm the PL. At this time, 110 days after the first collection, milk samples were also collected for SCC, bacteriological culture, and functional analysis of macrophages determined by flow cytometry.

First, for milk sampling, the strip cup test was performed to detect the presence of any obviously abnormal macroscopic alterations in milk. Afterwards, pre-dipping was performed and immediately after drying using a sheet of paper towel for each teat. After discarding the first three milk streams, the teat sphincter was scrubbed with 70% alcohol-moistened cotton, and individual quarter milk samples were aseptically collected in sterile flasks for microbiological analysis. Finally, milk samples were collected for SCC and evaluation of macrophage function and viability. The samples were kept at 4°C until arrival at the laboratory. Milk samples for bacteriological analysis were stored at −20°C for a maximum of 30 days until analysis.

Subsequently, each sample was coded and randomized, and the analyses were performed so that the researcher was not aware of the BLV status of the animal from which the sample was collected.

### Leukogram

The total leukocyte count was determined using an automated cell counter (ABX VET ABC, Horiba ABX Diagnostic®, Montpellier, France). The differential leukocyte count was performed using a routine blood smear.

### Bacteriological Examination of Milk

Bacteriological analyses were performed by culturing 10 μL of milk on 5% defibrinated sheep blood agar plates. The plates were incubated at 37°C for 72 h. For bacterial identification, macroscopic observation of colony morphology, Gram staining, and biochemical tests were performed (27). A milk sample was considered positive when there was the growth of one or more colonies (>100 CFU mL^−1^) (28, 29).

### Determination of the Somatic Cell Count

Milk samples for SCC determination were collected in 40-mL sterile flasks containing micropellets of the Bronopol preservative (2-bromo-2-nitropane-1,3-diol). Subsequently, SCC was performed with the automated somatic cell counter Somacount 300 (Bentley Instruments, Chaska, MN, USA).

### Separation of Milk Cells

The separation of milk cells was performed as described by Blagitz et al. (30, 31). In summary, 1 L of milk was diluted in 1 L of buffered saline solution (PBS). After centrifugation at 1000 × *g* for 15 min, the fat layer and the supernatant were discarded. The cell pellet was then washed again with 30 mL of PBS solution and centrifuged at 400 × *g* for 10 min. Subsequently, the cells were resuspended in 1 mL of RPMI-1640 cell culture medium (cat. n. R7638, Sigma Aldrich, USA) supplemented with 10% heat-inactivated fetal bovine serum (Cultilab, Brazil), and subsequently, the cells were counted in a Neubauer chamber. Cell viability was first assessed by trypan blue exclusion. The milk cells were then diluted with cell culture medium containing 10% fetal bovine serum at a concentration of 2 × 10^6^ mL^−1^ viable milk cells. Then, 100 μL of the milk cell suspension (containing 2 × 10^5^ cells) was transferred to tubes suitable for flow cytometry analysis for further flow cytometric analysis.

### Detection of Apoptosis by Flow Cytometry

Apoptosis of milk macrophages was determined by double staining with fluorescein isothiocyanate (FITC)-conjugated annexin-V and propidium iodide (PI) by flow cytometry analysis using a commercial kit (cat. n. K2350, APOPTEST-FITC, Dako Cytometrion, Netherlands), as previously described by Della Libera et al. (4) and Souza et al. (32). Initially, 2 × 10^5^ milk cells were resuspended in 100 μL of binding buffer (10 mM HEPES, 150 mM NaCl, 1 mM MgCl_2_, and 1.8 mM CaCl_2_) containing anti-annexin-V FITC antibody and incubated at room temperature for 20 min in the dark. Subsequently, the macrophages were labeled with a CD14-specific mAb, as described below. Immediately before the flow cytometry analysis, 5 μL of a 250 μg mL^−1^ PI solution was added. Cells negative for annexin-V FITC and PI were considered alive (Supplementary Figure 1). Cells that were reactive to FITC-labeled annexin-V but negative for PI were classified as apoptotic (Supplementary Figure 1). Finally, cells positive for both annexin-V FITC and PI were regarded as late apoptotic or necrotic (33) (Supplementary Figure 1).

The readings of the samples were performed using a FACSCalibur™ flow cytometer (Becton Dickinson Immunocytometry Systems™, San Diego, USA) with argon (excitation 488 nm) and diode (excitation 635 nm) lasers. Here, 20,000 cells, excluding most of the debris, were examined per sample. FlowJo software (Tree Star Inc., Ashland, USA) was used to analyze the data.

### Preparation of *Staphylococcus aureus* Stained With PI

The staining of heat-killed *S. aureus* (ATCC 25923) with PI was prepared as proposed by Hasui et al. (34) and slightly modified by Della Libera et al. (4).

### Intracellular Production of Rons

The intracellular production of RONS was performed by flow cytometry, as previously described (4, 31, 32), using 2′,7′ dichlorofluorescein diacetate (DCFH_2_-DA) as a probe. The various types of RONSs (hydrogen peroxide, peroxynitrite, nitric oxide, hydroxyl radicals, and peroxyl) oxidize DCFH_2_-DA into DCF, which is fluorescent and can be detected by flow cytometry (35). Briefly, 2 × 10^5^ viable milk cells were incubated with 200 μL of DCFH_2_-DA (0.3 mM, cat. n. D6883, Sigma Aldrich, St. Louis, USA) for 30 min at 37°C and 800 μL of PBS. Subsequently, 2 mL of 3 mM EDTA was added. Next, the macrophages were labeled with a CD14-specific mAb, as described below. Finally, the samples were centrifuged at 400 × *g* for 10 min, and the supernatant was discarded, and the leukocytes were resuspended in 300 μL of PBS.

Finally, 20,000 cells, except for most cellular debris, were examined per sample. The readings of the samples were performed using a FACSCalibur™ flow cytometer (Becton Dickinson Immunocytometry Systems™, San Diego, USA) with argon (excitation 488 nm) and diode (excitation 635 nm) lasers. FlowJo software (Tree Star Inc., Ashland, USA) was used to analyze the data. The data are presented as the percentage of macrophages (CD14^+^ cells; Supplementary Figure 1) that produced RONSs (percentage of fluorescent cells), and the geometric mean fluorescence intensity (GMFI) indicates the intensity of RONS production of each cell given by the measurement of fluorescence intensity. The results were corrected for autofluorescence content using non-stained milk cells from milk samples from the same mammary quarter.

### Phagocytosis

The phagocytosis assay was performed by flow cytometry using PI-conjugated *S. aureus*, as previously described (4, 32, 36). Briefly, 2 × 10^5^ viable milk cells were incubated with 100 μL of PI-conjugated *S. aureus* for 30 min at 37°C and 900 μL of PBS. Subsequently, 2 mL of 3 mM EDTA was added to drastically reduce the number of bacteria adhering to the cell membrane that could be mistakenly considered phagocytized (34, 37). Next, the macrophages were labeled with a CD14-specific mAb, as described below. Finally, the samples were centrifuged at 400 × *g* for 10 min, and the supernatant was discarded, and the leukocytes were resuspended in 300 μL of PBS.

Finally, 20,000 cells, excluding most cellular debris, were examined per sample. The readings of the samples were performed using a FACSCalibur™ flow cytometer (Becton Dickinson Immunocytometry Systems™, San Diego, USA) with argon (excitation 488 nm), and diode (excitation 635 nm) lasers. FlowJo software (Tree Star Inc., Ashland, USA) was used to analyze the data. The data are presented as the percentage of macrophages (CD14^+^ cells) that phagocytized PI-stained bacteria (percentage of fluorescent cells; Supplementary Figure 1), and the GMFI indicates the number of bacteria phagocytized per macrophage by measuring the fluorescence intensity, which is correlated with the number of phagocytized bacteria per cell. The results were corrected for autofluorescence content using non-stained milk cells from milk samples from the same mammary quarter.

### Identification of Milk Macrophages

Initially, the cells were incubated with 1 μL of mouse anti-bovine CD14 mAb (clone MM61A; VMRD Pullman, Pullman, USA) for 30 min at room temperature. Immediately after, 1 mL of PBS was added to the specific cytometry tube, and the samples were centrifuged at 400 × *g* for 8 min, and the supernatant was discarded. Subsequently, 1 μL of secondary antibody allophycocyanin-conjugated goat anti-mouse IgG1 (APC; cat. n. A10541, Invitrogen, Carlsbad, USA) was added to the samples, which were incubated for 30 min at room temperature. Then, 1 mL of the PBS solution was added to the cell suspension, which was centrifuged at 400 × g for 8 min, and the supernatant was discarded. The macrophages were identified using flow cytometry based on cells' CD14 positivity (Supplementary Figure 1). Finally, 300 μL of PBS was added to the samples that were analyzed by flow cytometry (BD FACSCalibur, Becton Dickinson Immunocytometry System™, San Diego, USA). The number of macrophages were determined by multiplying the percentage of overall macrophages (CD14^+^ cells) per the number of milk somatic cells per milliliter. To determine the percentage of macrophages (CD14^+^) among viable cells in quarter milk samples, dead cells (PI^+^) were excluded from the analysis. A negative control (unstained), fluorochrome-conjugated secondary antibody control, and single-stained samples were prepared for the compensation controls. FlowJo software (Tree Star Inc., Ashland, USA) was used to analyze the data.

### Statistical Analysis

The distributions of all variables were analyzed using normal probability plots obtained by the Shapiro and Wilk tests. As all data presented high coefficient of variation, we carried out a logarithmic transformation (Log_10_). First, interclass correlation at the cow and quarter levels was calculated to determine the strength of clustering, as previously described by McGraw and Wong (38). All variables presented ICC value >0.60 at both cow and udder quarter levels, apart from the percentage of apoptotic, late apoptotic/necrotic, and viable milk macrophages. The data were analyzed using ANOVA followed by the *post-hoc* Student–Newnan–Keuls test (39). The model of mammary quarters and cows nested within cows was considered (36). Statistical analyses were performed using the statistical software InfoStat (Cordoba, Argentina). The results are presented as the mean ± standard error. The significance level was set at *P* ≤ 0.05.

## Results

**Figure 1:**
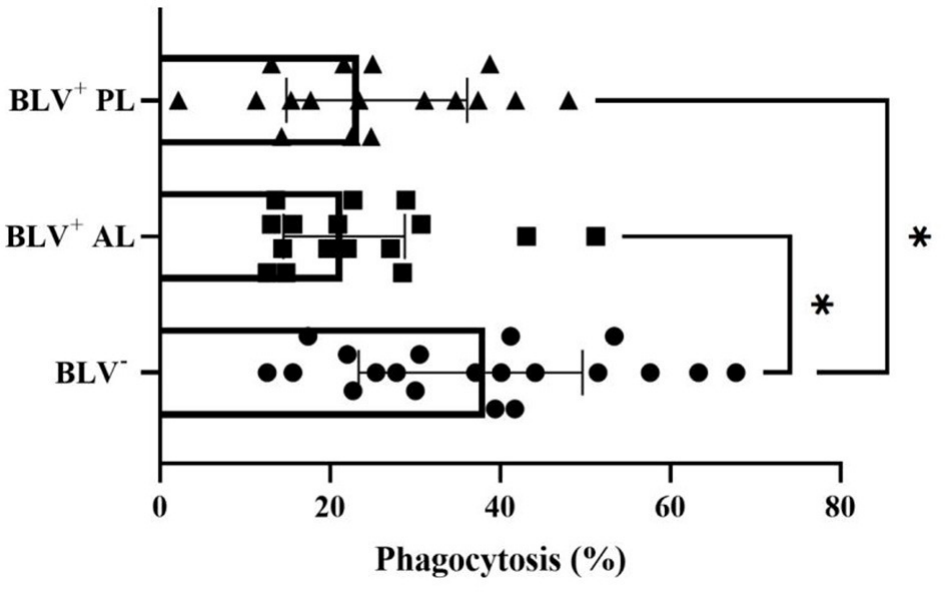
Percentage (individual values and median ± interquartile range) of milk macrophages that phagocytized *S. aureus* from health mammary glands in bovine leukemia virus (BLV) seronegative (BLV^−^) and in BLV-infected (BLV^+^) dairy cows without (AL) and with persistent lymphocytosis (PL). *P < 0.05.

The SCC, days in lactation, and parity (data not shown) values did not differ among groups. No effect of BLV infection in the overall number of macrophages per milliliter (*P* = 0.87) and in the percentage of viable macrophages (*P* = 0.09) among overall viable milk cells was found. In the present study, the percentage of milk macrophages that phagocytosed *S. aureus* in BLV-seronegative animals was higher than AL BLV-infected dairy cows (*P* = 0.008) (Figure 1) and those with PL (*P* = 0.015) (Figure 1). However, the percentage of milk macrophages that produced RONS (*P* = 0.22), the GMFI of intracellular RONS (*P* = 0.14), and the GMFI *S. aureus* phagocytosis (*P* = 0.79) did not differ among groups.

**Figure 2:**
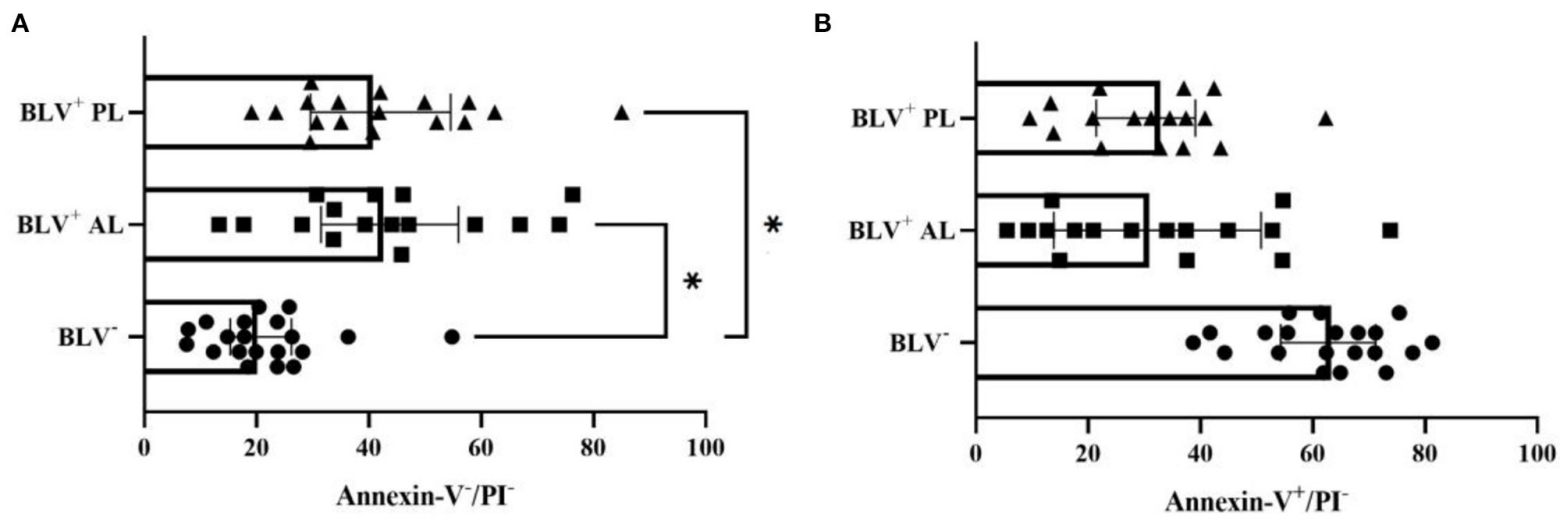
Percentage (individual values and median ± interquartile range) of viable (Annexin-V^−^/PI^−^) **(A)** and apoptotic (Annexin-V^+^/PI^−^) **(B)** milk macrophages from health mammary glands in bovine leukemia virus (BLV) seronegative (BLV^−^) and in BLV-infected (BLV^+^) dairy cows without (AL) and with persistent lymphocytosis (PL). *P < 0.03.

The AL BLV-infected dairy cows (Figure 2A) and those with PL (Figure 2A) had a higher frequency of viable milk macrophages (*P* = 0.03), while healthy animals had a tendency toward higher percentage of apoptotic milk macrophages (*P* = 0.06) (Figure 2B), although, no significant difference on the percentage of necrotic/late apoptotic milk macrophages was found (*P* = 0.48).

## Discussion

Although, most studies on BLV demonstrate its impact on the adaptive immune system, some studies show that BLV infection negatively affects the performance of monocytes (5, 13, 40); however, to the best of our knowledge, no study has investigated the impact of BLV infection on the function of milk macrophages. In this concern, although BLV persists mainly in B cells, monocytes and macrophages could be infected by BLV and act as a reservoir for BLV (1, 41).

Macrophages have a pivotal role in the initiation, maintenance, and resolution of inflammation (42). These cells represent the main leukocyte population in milk from mammary glands of healthy cows (21, 22, 24), in which the percentage of the milk macrophages found here was similar to the previous values reported by Sarikaya et al. (21, 22) and Takano et al. (24), but a little bit higher than others (43, 44). Therefore, macrophages are the first to find possible invasive pathogens. Macrophages play an important role in the defense of the mammary gland and, among various functions, they act on phagocytosis mechanisms, antigen presentation, lactoferrin synthesis, complement system factors, N-acetyl-β-D-glucosaminidase, and cytokines, in addition to removing cellular debris and apoptotic neutrophils (45–47).

Phagocyte's viability and their capacity to produce ROS and phagocytosis bacteria are inextricably connected. However, even though we expected a higher macrophage functionality in milk quarter samples with a higher percentage of viable cells, BLV infection impaired *S. aureus* macrophage phagocytosis while having a higher frequency of viable macrophages. Similarly, we have shown a reduction in the percentage of *S. aureus* phagocytosis by blood monocytes in cows infected with BLV with PL (5). Furthermore, we also have demonstrated that monocytes from cows with PL showed a lower phagocytosis of zymosan particles of *Saccharomyces cerevisiae* than both BLV-seronegative and AL BLV-infected dairy cows (13). Therefore, although BLV infection affects the phagocytic capacity of blood monocytes only in animals with PL, our findings showed that the function of milk macrophages is impaired in BLV-infected animals regardless of the manifestation of PL. Furthermore, Werling et al. (48) showed that CD14^+^ blood monocytes from BLV-infected dairy cows had less Fcγ-receptor mediated phagocytosis, which could, at least in part, explain our results. Although, regarding our outcomes, it appears that this dysfunction is preserved in milk macrophages, it needs to be further investigated.

Although, the mechanism by which BLV interferes with milk macrophage apoptosis is unknown, we assume that inhibition of milk macrophage apoptosis could be related to the intracellular levels of glutathione that is correlated with BLV-associated protection against apoptosis, which could be linked to the decrease of oxidation and subsequent impairment of apoptosis (49), although, this mechanism in milk macrophages needs to be further elucidated. Furthermore, we also hypothesized that the higher percentage of milk macrophages in BLV-infected cows with PL may be associated, at least in part, with the expression of the *Bcl-2* and *Bcl2 L1* genes involved in apoptotic inhibition (48), in line with the higher viability of milk macrophages found here in BLV-infected dairy cows. Although, it is not yet known exactly how BLV alters cell death and growth mechanisms, there is evidence to suggest that BLV can affect signaling pathways for cell growth and death (2, 40).

Overall, our results indicate that BLV infection may favor alternatively activated macrophages (M2 macrophages), as lower *S. aureus* phagocytosis was found, but this hypothesis needs to be further investigated. In this context, M2 macrophages was associated with lower phagocytosis of bacterial pathogens (50, 51). Furthermore, the lower frequency of apoptotic milk macrophages could be also explained by higher efferocytosis capability of M2 macrophages (52) in BLV-infected dairy cows, which lead to the phagocytic removal of apoptotic cells by the recognition of the “eat me” signals (e.g., exposure of phosphatidylserine) from cells undergoing apoptosis (52, 53). In fact, BLV was associated with IL-10 production by peripheral blood mononuclear cells (54), which, in turn, favors M2 polarization (53). Additionally, M2 macrophages were associated with a worse clinical prognosis of adult T-cell leukemia/lymphoma caused by human T-cell leukemia virus type 1 (55), a virus closely related to BLV (2). Indeed, viruses usually evolve mechanisms to enhance M2 macrophages, which exert potent immunosuppressive effects (56).

Furthermore, we should regard that our macrophage activities were carried out in the context where macrophage activities could be modified by other cell types, as all milk cells are present in functional assays. Similar to our study, many studies have used all milk cells in their assays to investigate activities of a particular cell type (4, 29–33, 36), as have long been performed with blood cells (so-called whole blood assay), which has largely been used and regarded as simple, reproducible, and clinically relevant, allowing us to *ex vivo* assess immune functions (57, 58).

An important aspect that could affect our outcomes is the interdependence of udder quarters and the fact that some quarters had IMIs, which could be a bias of our data analysis due to the effect of IMIs on immune response of the healthy neighborhood mammary gland quarters (4). In this concern, to deal with it, as the immune response between quarters within cow is not an independent process (36, 59), we considered it in the statistical analysis by determining the intraclass correlation (60), and using a model of udder quarters and cows nested with cows (36), as previously proposed.

Therefore, despite the limited number of animals used in the present study, our findings demonstrate that BLV infection can negatively affect the immunity of the mammary gland, which could predispose animals to coinfections or superinfections and can augment the severity of infections, as previously described for bovine udder health (4, 15, 16, 61, 62). Thus, it is clear that BLV infection can impact the phagocytic capacity of macrophages against mastitis-causing pathogens (e.g., *S. aureus*) and may favor susceptibility to infections or decrease their ability to eliminate intramammary infection (spontaneous cure). Thus, our study strengthens the idea that the impact of this chronic disease with low lethality is underestimated due to its association with comorbidities.

## Conclusion

The present study provides crucial information on the implications of BLV infections in the mammary gland and negatively impacts the functionality of milk macrophages in BLV-infected animals. In addition, this study highlights the importance of controlling BLV infections due to their indirect effects on the emergence of secondary infections, such as mastitis.

## Data Availability Statement

The raw data supporting the conclusions of this article will be made available by the authors, without undue reservation.

## Ethics Statement

The animal study was reviewed and approved by Bioethic Commission of the Faculty of Veterinary Medicine and Animal Science of University of São Paulo. Written informed consent was obtained from the owners for the participation of their animals in this study.

## Author Contributions

EL drafted and edited the manuscript. MB performed the experiments and designed the studies. CB participated in flow cytometry analysis. AA, AF, HF, and ER provided technical help and edited the manuscript. AD designed and supervised the studies. FS performed the analysis, designed the studies, and edited the manuscript. SD and MS performed all statistical analysis and edited the manuscript. All authors have read and agreed to the published version of this manuscript.

## Conflict of Interest

The authors declare that the research was conducted in the absence of any commercial or financial relationships that could be construed as a potential conflict of interest.

## Supplementary Material

Supplementary Figure 1 Successive gating scheme for assessment the functional analysis of milk macrophages (CD14^+^ cells). Recording of scatter and fluorescent properties were performed in a standard population excluding most cell debris **(A)**. Then, the CD14^+^ cells (macrophages) were analyzed based on their cytoplasmic granularity and mean fluorescence intensity following a two-step fluorescent immunolabeling protocol using primary anti-bovine monoclonal antibody (Ab) specific for CD14^+^ cells identification and secondary Abs coupled to long-wavelength fluorescent probe [FL4-H; **(B)**]. Afterwards, the percentage of CD14-positive cells that intracellularly produced reactive oxygen species **(C)** and phagocytosed *Staphylococcus aureus* **(D)** were determined. Furthermore, annexin-V-fluorecein isothiocynate (FL1-H) vs. propidium iodide (FL3-H) dot plot was performed to assess the percentage of apoptotic (Q1) and viable (Q4) CD14-positive events in milk **(E)**.
