# Supplementary figures and images for: Milk Macrophage Function in Bovine Leukemia Virus-Infected Dairy Cows
Source: Front Vet Sci. 2021 Jun 17;8:650021. doi: 10.3389/fvets.2021.650021 (PMC8245700; doi:10.3389/fvets.2021.650021)

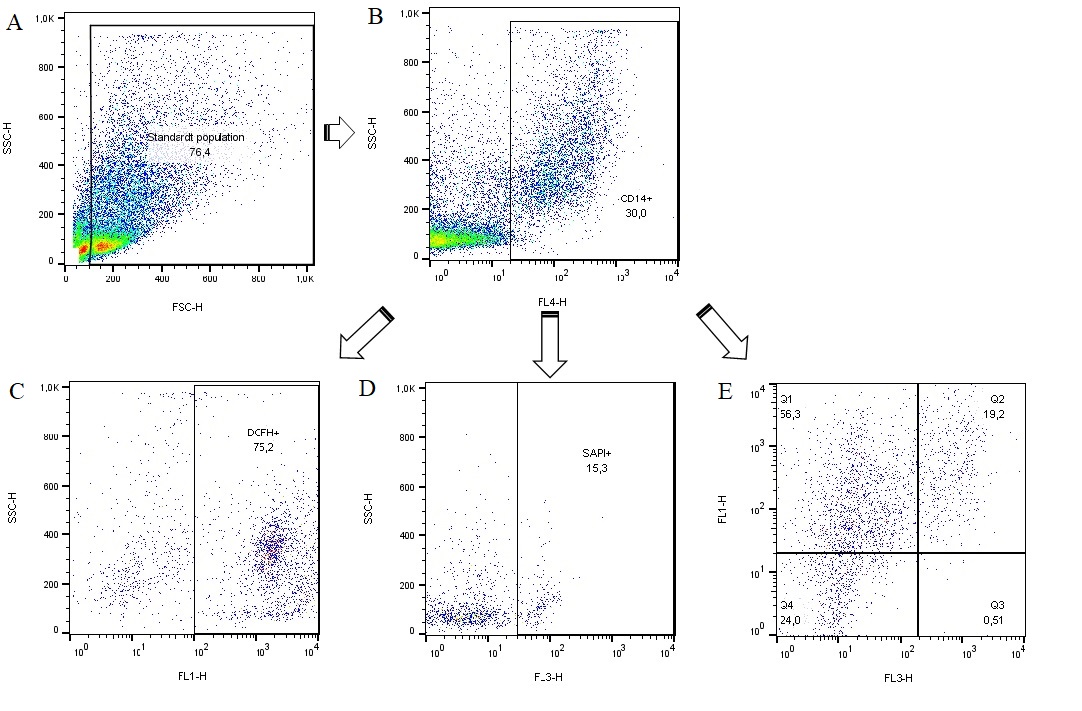

Supplement: Supplementary Figure 1 — Successive gating scheme for assessment the functional analysis of milk macrophages (CD14+ cells). Recording of scatter and fluorescent properties were performed in a standard population excluding most cell debris (A). Then, the CD14+ cells (macrophages) were analyzed based on their cytoplasmic granularity and mean fluorescence intensity following a two-step fluorescent immunolabeling protocol using primary anti-bovine monoclonal antibody (Ab) specific for CD14+ cells identification and secondary Abs coupled to long-wavelength fluorescent probe [FL4-H; (B)]. Afterwards, the percentage of CD14-positive cells that intracellularly produced reactive oxygen species (C) and phagocytosed Staphylococcus aureus (D) were determined. Furthermore, annexin-V-fluorecein isothiocynate (FL1-H) vs. propidium iodide (FL3-H) dot plot was performed to assess the percentage of apoptotic (Q1) and viable (Q4) CD14-positive events in milk (E). [file Image_1.JPEG]
